# Coronary Artery Bypass Grafting on Microaxial Flow Pump Support in Patients With Severely Reduced Left Ventricular Ejection Fraction

**DOI:** 10.1111/aor.15038

**Published:** 2025-06-04

**Authors:** Gaik Nersesian, Daniel Lewin, Yuriy Hrytsyna, Pia Lanmueller, Sascha Ott, Nicolas Merke, Volkmar Falk, Felix Schoenrath, Evgenij Potapov, Alaa Abd El Al

**Affiliations:** ^1^ Department of Cardiothoracic and Vascular Surgery Deutsches Herzzentrum der Charité (DHZC) Berlin Germany; ^2^ DZHK (German Centre for Cardiovascular Research) Partner Site Berlin Berlin Germany; ^3^ Department of Cardiac Anesthesiology and Intensive Care Medicine Deutsches Herzzentrum der Charité (DHZC) Berlin Germany; ^4^ Berlin Institute of Health at Charité—Universitätsmedizin Berlin Berlin Germany; ^5^ Department of Health Sciences and Technology Institute of Translational Medicine, Translational Cardiovascular Technologies, Swiss Federal Institute of Technology (ETH) Zurich Zurich Switzerland

**Keywords:** coronary artery bypass grafting, coronary artery disease, Impella, microaxial flow pump, reduced left ventricular ejection fraction

## Abstract

**Introduction:**

Patients with coronary artery disease (CAD) and severely reduced left ventricular ejection fraction (LVEF) face high perioperative risks during surgical revascularization. This case series examines outcomes in CAD patients with LVEF ≤ 25% undergoing surgical revascularization on microaxial flow pump (mAFP) support.

**Methods:**

We retrospectively analyzed 12 patients at Deutsches Herzzentrum der Charité who underwent scheduled protected coronary artery bypass grafting (CABG) with full‐flow mAFP support. Patients with acute myocardial infarction or no myocardial viability were excluded.

**Results:**

The cohort had a median age of 60 years [59; 66], 92% male, BMI 26 ± 6.2 kg/m^2^, median LVEF 18% [15; 24], and LVEDD 69 mm [59; 78]. Seven patients had diabetes mellitus and chronic renal failure, and five had prior myocardial infarctions. The mean EUROSCORE II was 2.5 ± 0.6.

Surgical revascularization was performed with ongoing mAFP support, with a median of 3 distal anastomoses. Complete revascularization was achieved in 11 cases and surgical time was 254 min [187; 266]. Myocardial recovery occurred in seven patients, while four required durable left ventricular assist device implantation, and one died on mAFP support. Two (16.6%) patients died during a follow‐up period of 93 days. Median Impella support lasted 6 days [3; 9], invasive ventilation 13 h [11; 20], and ICU stays 4.5 days [4; 17].

Complications included one bleeding requiring revision, two mAFP exchanges due to thrombosis/dislodgement, and four thromboembolic strokes in three patients during mAFP explantation/exchange.

**Conclusion:**

Revascularization with mAFP support is a feasible approach for high‐risk CAD patients but is associated with support‐related complications, including thromboembolic strokes during mAFP manipulations (e.g., explantation or exchange). Prospective randomized trials are essential to evaluate the potential benefits of intraoperative mAFP support during surgical revascularization compared to alternative mechanical support strategies and/or pharmacological measures.

## Graphical abstract

Coronary artery revascularization with microaxial flow pump support in patients with severely reduced left ventricular ejection fraction is an emerging technique in cardiac surgery. In this manuscript, we present early results, highlight the advantages, and discuss the key limitations of this approach.

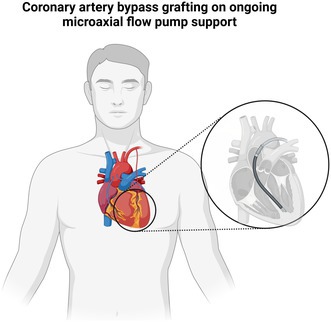

## 1 Introduction

Patients with coronary artery disease (CAD) and reduced left ventricular ejection fraction (LVEF) represent a high‐risk population with an increased mortality and morbidity [1]. Therapy for those patients is challenging and complex and should address both structural heart disease and heart failure.

Revascularization either with percutaneous coronary interventions (PCI) or coronary artery bypass grafting (CABG) represents a valuable option for those patients and may not only improve coronary perfusion but also protect from future myocardial infarctions [2]. Recent studies on CABG in patients with reduced LVEF are mainly focused on patients with LVEF of 35% and higher [3, 4]. Thus, very limited data about patients with LVEF ≤ 25% is available; thus, this population can be also considered for a durable left ventricular assist device implantation (LVAD) [5, 6].

Percutaneously implanted mAFP (Impella, Abiomed, Danvers, MA, USA) has been successfully used for high‐risk interventional coronary interventions [7, 8]. However, limited data is available on the utilization of full‐support mAFP in patients undergoing surgical revascularization [9]. To close the knowledge gap and to stratify which patients benefit from surgical revascularization, we aim to describe the effect of full support mAFP in patients with LVEF ≤ 25% undergoing surgical revascularization.

## 2 Patients and Methods

### 2.1 Ethics Statement

The presented study was approved by the institutional ethics committee of the Charité Universitaetsmedizin Berlin (EA4/108/24). All medical records were retrospectively reviewed and the requirement for informed consent was waived.

### 2.2 Patient Selection

Elective patients with 3‐vessel disease and no recent (< 7 days) myocardial infarction admitted to our institution underwent an echocardiographic evaluation to assess myocardial contractility and valvular pathologies. Patients with a CAD and severely reduced LVEF underwent a preoperative myocardial viability assessment in order to evaluate the amount of scar tissue in the targeted anastomosis areas. Regions with a scar tissue amount ≥ 75% and no viability were considered unsuitable for surgical revascularization.

Myocardial viability assessment was preferably performed with a gadolinium‐enhanced cardiac MRI. Alternatively, in patients with a contraindication for MRI, positron emission tomography cardiac computed tomography (PET‐CT) imaging or myocardial scintigraphy CT was performed. During preoperative assessment, patients simultaneously underwent necessary diagnostics for a dLVAD evaluation (including gastroscopy, coloscopy, full‐body CT scan and required medicals consults).

Twelve consecutive patients with CAD, LVEF ≤ 25% who demonstrated viable myocardium in coronary target areas were selected for CABG on mAFP support. Preoperatively, patients were treated with at least four guideline‐directed heart failure drugs at the maximum tolerated dose to improve symptoms and secondary organ function. Patients required no inotropic support prior to the surgery.

### 2.3 Surgical Technique

For Impella 5.5 implantation, 250 IU of heparin per kg body weight were administered aiming for a target ACT of > 300 s as per standard of off‐pump coronary artery bypass grafting.

The right axillary artery was surgically exposed and a 10‐mm Hemashield graft (MAQUET Ltd., Rastatt, Germany) was anastomosed end‐to‐side and tunneled under the skin to allow primary wound closure. The Impella 5.5 mAFP was inserted through the graft under fluoroscopic and echocardiographic guidance. The inlet was positioned approx. 4.5 cm below the level of the aortic valve annulus. After optimal positioning of the pump was achieved, the highest speed level possible was set during the surgery with a target mean arterial pressure (MAP) of −60–70 mmHg.

The surgical coronary revascularization was performed on mAFP support in a beating heart technique, with cardiopulmonary bypass as a back‐up. In none of the patients was initiation of cardiopulmonary bypass or any additional mechanical circulatory support other than the Impella mAFP (e.g., due to hemodynamic instability or concomitant procedures) required during the procedure. An Acrobat‐I stabilizer (Maquet/Getinge Group, Göteborg, Sweden) was used, along with temporary coronary occlusion sutures or Axis coronary shunts (Maquet/Getinge Group, Göteborg, Sweden). During the positioning of the stabilizer for distal anastomoses, the depth of the mAFP was constantly monitored by transesophageal echocardiography, and repositionings were performed if necessary to avoid LV mechanical damage or pump dislodgement.

For proximal anastomosis, the ascending aorta was either partially clamped using a tangential clamp or a HEARTSTRING device (Maquet/Getinge‐Group, Göteborg, Sweden) was applied. Special care was taken not to damage or accidentally fixate the mAFP catheter in the ascending aorta.

**FIGURE 1:**
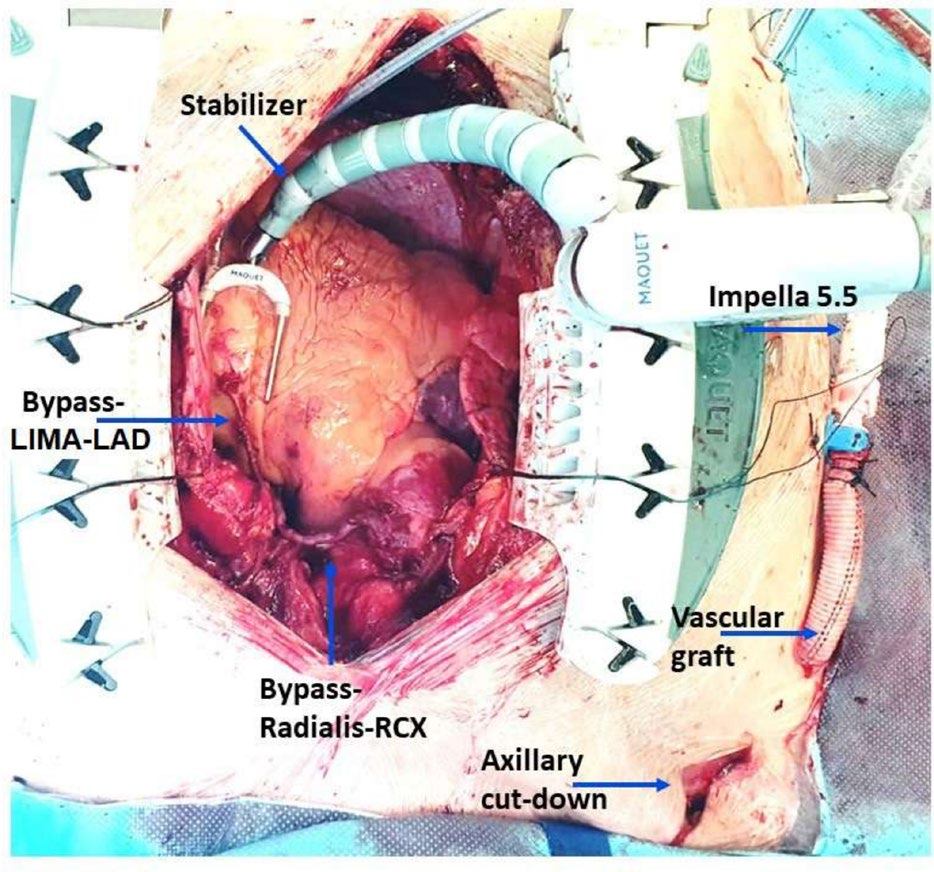
LAD, left anterior descending artery; LIMA, left internal mammary artery; RCx, ramus circuflexus of the left coronary artery. [Color figure can be viewed at wileyonlinelibrary.com]

**FIGURE 2:**
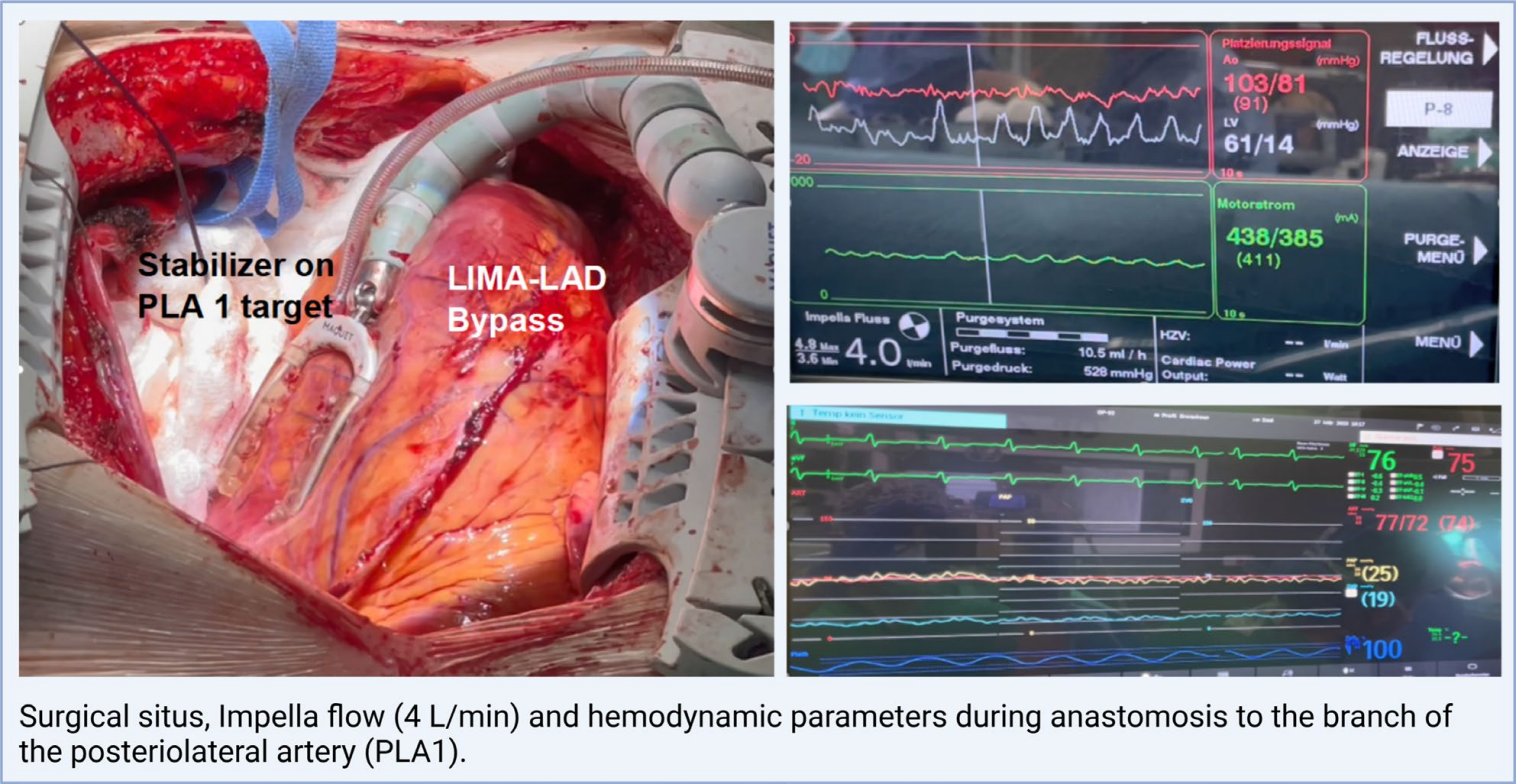
LAD, left anterior descending artery; LIMA, left internal mammary artery; PLA, posterior lateral artery. [Color figure can be viewed at wileyonlinelibrary.com]

**FIGURE 3:**
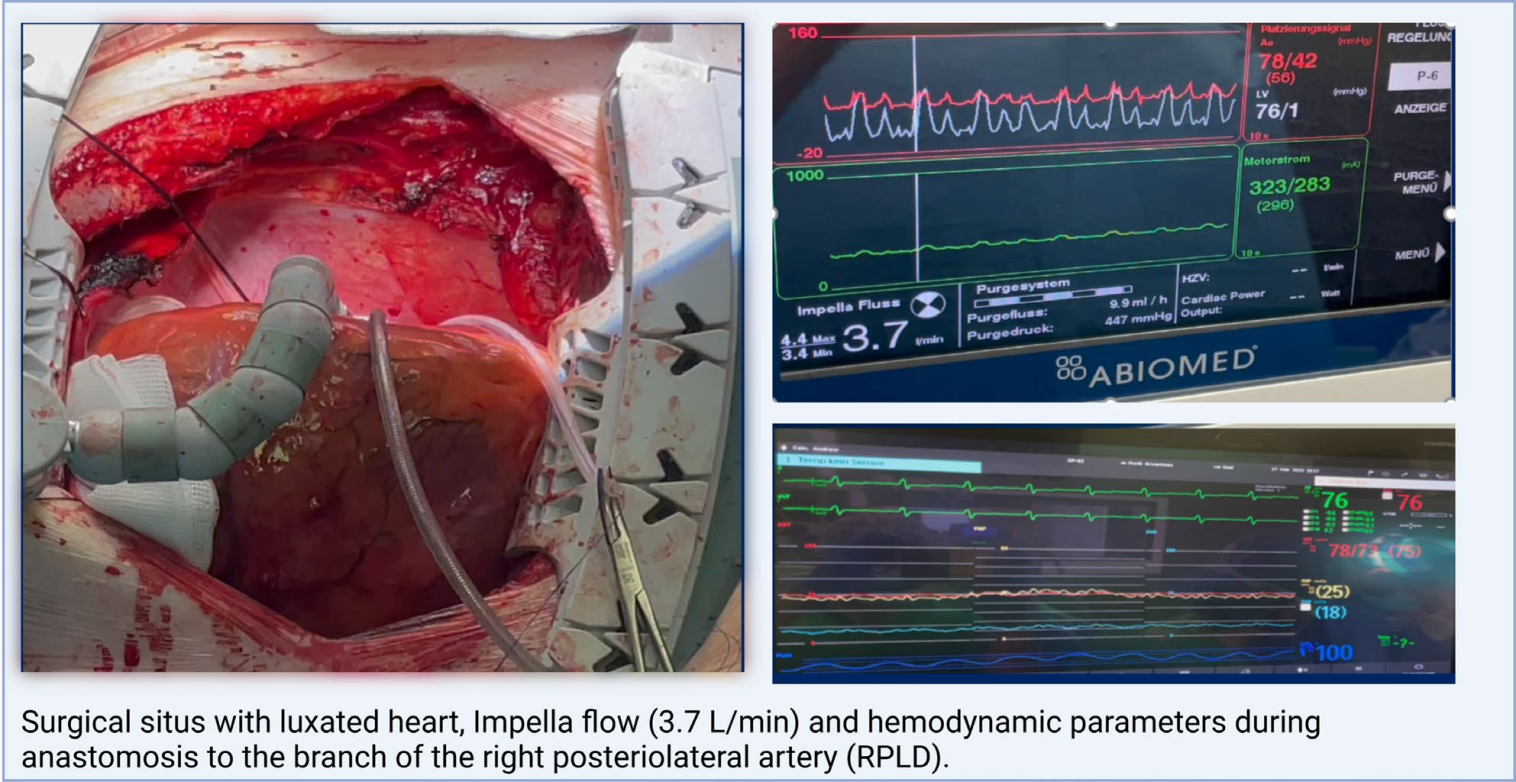
RPLD, right posteriorlateral artery. [Color figure can be viewed at wileyonlinelibrary.com]

Surgical situs with hemodynamic parameters and position of the stabilizer for different coronary targets are shown in Figures 1, 2, 3.

In all patients, an anticoagulant‐free, dextrose‐ and bicarbonate‐based purge solution was used for mAFP. At our institution, postoperative anticoagulation is routinely managed with continuous intravenous administration of unfractionated heparin, targeting an aPTT of 50–55 s. Heparin administration is initiated 4–6 h after the patient's arrival in the ICU. In cases of heparin‐induced thrombocytopenia (HIT II), anticoagulation is switched to argatroban.

Weaning from mAFP was conducted according to our institutional weaning protocol. In patients achieving inotropic‐free status (or requiring only low‐dose vasopressors) the mAFP support has been gradually reduced down to P2 level under meticulous echocardiographic evaluation [10]. If weaning was successful, mAFP was removed bedside under local anesthesia. In cases when weaning from tMCS was not possible, durable LVAD implantation was carried out [10].

### 2.4 Calculations

The MELD‐XI score was calculated using the formula: 5.11 × ln(bilirubin) + 11.79 × ln(creatinine) + 9.44 [11].

The LVESV index was calculated using the formula:

LVESV/BSA [12] (left ventricular endsystolic volume/body surface area).

## 3 Results

All patients received mAFP intraoperatively prior to coronary anastomoses. Distal and proximal aortocoronary anastomoses were performed in off‐pump beating heart technique on running mAFP, which was continued after the completion of the surgery for the postoperative support.

Relevant demographic, laboratory and echocardiographic parameters are listed in Tables 1, 2, 3 respectively (Table 1. *Preoperative demographics*, Table 2. *Laboratory parameters*, Table 3. *Echocardiographic parameters*).

**TABLE 1:** Patient demographics.

Patient Nr.	Age	Sex	BMI	BSA	AMI	Type of AMI	COPD	AHT	AF	DM	IDDM	CKD	CKD Stage	HLP	PAD	STS Score	ES II
1	57	m	29.9	2.38	1	NSTEMI	0	1	0	1	0	1	3	1	0	1,4	2,27
2	76	m	36.7	2.16	1	NSTEMI	1	1	1	1	0	1	2	1	1	4,85	3,17
3	55	m	39.4	2.50	0	0	0	1	0	1	0	1	3	0	0	3,93	3,45
4	58	m	20.9	1.96	0	0	0	1	0	1	0	0	0	1	0	1	1,69
5	59	m	22.9	1.96	1	NSTEMI	1	0	0	0	0	1	2	0	0	1,24	3,17
6	65	m	29.7	2.12	0	0	0	1	0	0	0	1	2	1	0	1,23	2,61
7	64	m	23.1	1.84	1	NSTEMI	0	1	0	1	0	0	0	1	0	1,8	1,89
8	60	m	21.6	2.11	0	0	0	1	1	0	0	0	0	0	0	1,63	1,69
9	65	m	21.1	1.80	0	0	0	1	0	1	0	1	2	0	0	2,37	2,61
10	75	f	21.2	1.32	0	0	1	1	0	0	0	0	0	0	0	3,6	3,37
11	70	m	24.0	1.74	0	0	0	1	0	1	0	1	2	0	0	1,56	2,65
12	63	m	21.9	1.6	1	NSTEMI	0	1	0	0	0	0	0	1	0	4,2	1,88

Abbreviations: AF, atrial fibrillation; AHT, arterial hypertension; AMI, acute myocardial infarction; BMI, body mass index; BSA, body surface area; CKD, chronic kidney disease; COPD, chronic obstructive pulmonary disease; DM, diabetes melitus (Type I); ES II, euroscore II; f, female; HLP, hyperlipoproteinemia; IDDM, insulin dependent diabetes melitus (Type I); m, male; (N)STEMI, (non) ST‐elevation myocardial infarction; PAD, peripheral arterial disease; STS, society of thoracic surgeons.

**TABLE 2:** Preoperative laboratory parameters.

Parameter	Value
pH	7.4 ± 0.06
Potassium (mmol/L)	4 ± 0.3
Sodium (mmon/L)	139 ± 3.1
Lactate (mmol/L)	0.9 ± 0.4
Hemoglobin	14 ± 2
Hematocrit	42.5 ± 6.4
WBC K/μL	8.1 ± 4.1
PLT K/μL	209 (195; 251)
CRP	0.65 (0.38; 1.1)
Albumin	4.1 ± 0.4
aPTT	37 (34; 39)
INR	1.1 ± 0.1
CK	77 (45; 116)
CK‐MB	17 (15; 20)
GOT	27 ± 4
GPT	24 ± 7.6
GGT	58 (30; 100)
LDH	182 (153; 204)
Lipase	43 (26; 61)
Creatinine	1.1 ± 0.3
GFR	72 (64; 95)
Urea	40 (32; 45)
Total Bilirubin	0.6 ± 0.2
MELD‐XI score	10.2 ± 1.2

Abbreviations: aPTT, activated partial thromboplastin time; CK, creatine kinase; CK‐MB, creatine kinase myocardial type; CRP, C reactive proteine; GFR, glomerular filtration rate; GGT, gamma‐glutamyl transferase; GOT, glutamate oxaloacetate transaminase; GPT, glutamat‐pyruvat‐transaminase; INR, international normalized ratio; LDH, lactate dehydrogenase; MELD, Model for End‐Stage Liver Disease; PLT, platelets; WBC, white blood cells.

**TABLE 3:** Preoperative echocardiography.

Patient Nr.	LVEF (%)	RVEF (%)	RV SV (mL)	LVEDD (mm)	LVESD (mm)	LVEDV (mL)	LVESV (ml)	Index LVESV (mL/m^2^)	GLS	Valves
1	20	48	63	71	64	290	232	97	−5	MI I; TI I
2	15	35		83	74	463	396	183	−2,1	MI II, TI II
3	24	31	74	82	72	378	286	114	−6,6	MI II; TI III
4	16	37	59	59	43	219	176	90	−3,4	MI I
5	15	37	89	76	69	370	317	162	−2,3	MI II, TI II
6	19	34	37	67	56	199	155	73	−4,6	MI II; TI I
7	13	32	69	77	72	367	320	174	−3,5	MI II; TI I
8	15	51	50	84	77	311	252	119	−4,6	MI II; TI I
9	14	45	49	53	48	215	184	102	−5,6	MI I; TI I
10	25	50		59		185	136	103	−7,3	MI I; TI I; AI I
11	20	45	60	66	60	280	234	80	−5.2	MI I; TI I
12	15	40	65	74	68	360	305	98	−3.6	MI I; TI I

Abbreviations: AI, aortic insufficiency; GLS, global longitudinal strain; L(R)VEF, left (right) ventricular ejection fraction; LVEDD, left venrticular enddiastolic diameter; LVESD, left ventricular endsystolic diameter; MI, mitral insufficiency; RV SV, right ventricular stroke volume; TAPSE, tricuspid annular plane systolic excursion; TI, tricuspid insufficiency.

### 3.1 Outcomes

Successful coronary revascularization on mAFP support, with anastomosis of all intended targets, was achieved in each case (Table 4. Surgical data and outcomes). No intraoperative complications, such as perforation or damage to the valvular apparatus or myocardium during mAFP implantation, were observed.

**TABLE 4:** Surgical data and outcomes.

Patient Nr.	Anastomoses performed	Surgery time (min)	VIS 24 h	Blood products use on mAFP	mAFP duration (d)	Duration of ventilation (h)	ICU duration (d)	In‐hospital stay (d)	Outcome	Discharged to
1	LIMA‐D1, VSG‐PLA, VSG‐RCA	257	1	0	1	8,5	2	6	Recovery— > Impella weaning	Other hospital/home
2	VSG‐ LAD, VSG‐RPLS	290	81	RBC 10 FFP 10 PC 10	4	95	4	11	Death	Death
3	LIMA‐ LAD, VSG‐PLA, VSG‐RPLS‐PDA	341	6	RBC 7 FFP 6 TK 2	5	17	5	18	Recovery— > Impella weaning	Other hospital/home
4	LIMA‐D1, VSG‐RIVA, VSG‐M1‐PDA	270	0	RBC 2 FFP 2	8	8	4	34	Recovery— > Impella weaning	Other hospital/home
5	LIMA‐ LAD, Radialis‐D1‐PLA, VSG‐PDA‐RPLD	264	20	RBC 11 FFP 2 PC 4	10	64	17	32	No Recovery— > LVAD	Other hospital/home
6	VSG‐D1‐ LAD, VSG‐ RPLS	259	9	RBC 4 FFP 3	8	24	17	43	No Recovery— > LVAD	Other hospital/home
7	LIMA‐ LAD, VSG‐ RPLS	188	0	PC 2	9	19	81	93	Recovery— > Decompensation— > LVAD	Death
8	LIMA‐ LAD, VSG‐PLA‐RPLS	174	0	0	23	10	29	29	No Recovery— > LVAD	Other hospital/home
9	LIMA‐ LAD, VSG‐ RPLS, VSG‐PDA	190	0	0	10	13	9	12	Recovery— > Impella weaning	Other hospital/home
10	LIMA‐ LAD, VSG‐PLA, VSG‐RPLS	251	0	RBC 4 FFP 1	1	11	2	6	Recovery— > Impella weaning	Other hospital/home
11	LIMA‐LAD, VSG‐RPLS	177	0	0	4	12	3	8	Recovery— > Impella weaning	Other hospital/home
12	LIMA‐LAD, VSG‐RCA, Radialis‐PLA	184	0	RBC 4 FFP 4	3	11	4	18	Recovery— > Impella weaning	Other hospital/home

Abbreviations: CX, circumflexus artery; FFP, fresh frozen plasma; ICU, intensive care unit; LAD (D1), left anterior descending artery; LIMA (D1), left internal mammary artery; LVAD, left ventricular assist device; mAFP, microaxial flow pump; NA, non applicable; PC, platelet concentrate; PDA, posterior descending artery; PLA, posteriolateral artery; RBC, red blood cell concentrate; RCA, right coronary artery; RIM, ramus intermedius; RIMA, right internal mammary artery; RPLS(D), ramus posteriolateralis sinister (dexder); VIS, vasoactive inotropic score; VSG, vena saphena granda.

Seven patients were successfully weaned from mAFP support after a median duration of 4.2 days. Three patients underwent durable LVAD implantation after a median of 5 days on mAFP. Patient No. 7 was initially weaned from mAFP but deteriorated 2 days later, requiring durable LVAD implantation. Patient No. 7 died due to septic multiorgan failure 70 days after surgery. Patient No. 2 developed severe respiratory failure due to SARS‐CoV‐2 infection, resulting in combined septic and cardiogenic shock. Despite ongoing mAFP support, peripheral va‐ECMO implantation became necessary. No significant clinical improvement was achieved, and the patient died on postoperative Day 4.

Eleven patients achieved the 30‐day survival benchmark, and ten were discharged home.

### 3.2 Complications

Four episodes of thromboembolic strokes in three patients were observed after mAFP manipulation (explantation/exchange); recanalization was performed in two cases. In two cases, residual neurological symptoms were present at discharge. Exchange was necessary in two patients for pump dislodgement and pump thrombosis, respectively. Access site bleeding requiring intervention occurred in one patient; another patient presented a postoperative peripheral neural lesion of the right arm, which was completely regredient by the time of discharge. Postoperative renal replacement therapy was necessary in one patient. In none of patients was an mAFP graft infection observed.

Re‐thoracotomy for bleeding was necessary in two patients; bleeding sources were the intercostal artery and proximal anastomosis, respectively.

## 4 Discussion

In this study, we demonstrated that surgical revascularization on full‐flow mAFP support in patients with a CAD and severely reduced LVEF is a feasible procedure, but is associated with the risk of thromboembolic and access‐related complications. The tMCS with mAFP enables the performance of CABG in high‐risk patients and bridges them to subsequent treatment in cases of persistent myocardial dysfunction despite revascularization. However, indications for mAFP application in the setting of protected CABG should be carefully weighed against the risk of therapy‐associated complications.

The first‐line treatment for patients with ischemic heart failure is optimal guideline‐directed medical and device therapy (OMDT), which must be constantly re‐evaluated and adjusted based on the patient's clinical condition. Coronary revascularization represents the next therapeutic step and may be considered for selected patients [13]. In recent years, the use of calcium sensitizers in cardiac surgery has increased. Prophylactic perioperative administration of calcium sensitizers in CABG patients with reduced LVEF has shown potential benefits in preventing postoperative LCOS and reducing mortality compared to placebo. However, the effectiveness of calcium sensitizers compared to standard LCOS therapy remains limited [14, 15]. Notably, current studies on prophylactic calcium sensitizer use have primarily included patients with LVEF < 40% (some with LVEF < 30%), whereas our study population had a median LVEF of 18%, representing a significantly different patient population [14]. The effectiveness of calcium sensitizers in such patients has yet to be investigated. It is important to note that calcium sensitizers were not used in the patients included in this study.

In heart failure patients who exhibit persistent symptoms despite OMDT and in whom revascularization did not improve the clinical condition or was not indicated, durable left ventricular assist device implantation or heart transplant represents the ultimate treatment [6, 16].

### 4.1 Indication for Revascularization

The decision‐making process in patients with CAD and severely reduced LVEF is complex and should involve a heart team approach to indicate the optimal treatment strategy. In this context, cardiac imaging for ischemia and myocardial viability assessment serves as a viable diagnostic tool for preoperative evaluation.

Standard LVEF measurement is a well‐established parameter for assessing cardiac function. However, in patients with dysfunctional myocardial contractility and/or mitral valve regurgitation, LVEF may underestimate the severity of functional impairment [12]. At our institution, a strain echocardiography is routinely performed in patients with advanced heart failure for a more precise evaluation of therapy planning [12].

Modern imaging modalities, such as contrast‐enhanced magnetic resonance imaging (MRI) and positron emission tomography (PET) enable the visualization of myocardial viability by detecting the uptake and retention of metabolically active tracers [17]. Preoperative myocardial viability assessment is a sophisticated diagnostic tool for the indication for revascularization. However, evidence regarding the clinical benefit of viability‐driven revascularization remains limited [18, 19].

In case of patients with multi‐vessel disease and reduced LVEF, there is ongoing controversy regarding the optimal revascularization strategy. The results of the REVIVED‐BCIS2 trial, which investigated whether percutaneous coronary intervention (PCI) can improve outcomes in patients with ischemic HF compared to the guideline‐directed OMDT alone, did not demonstrate a significant survival benefit. The two‐year all‐cause mortality rates were 31.7% for the PCI cohort and 32.6% for the OMDT group [20]. Although the overall prevalence of AMI was similar between the groups, at 10.7% and 10.8% respectively, nearly 40% of AMIs in the PCI cohort occurred as periprocedural complications, which may explain the neutral outcomes of the study [20].

In contrast, the STICH trial (Surgical Treatment for Ischemic Heart Failure) suggested a significant reduction in mortality and HF‐related hospitalizations over a follow‐up period of up to 10 years [21]. Similarly, the ISCHEMIA trial demonstrated comparable results in patients with stable angina and moderate‐to‐severe ischemia on diagnostic assessment, who received OMGT with and without revascularization (CABG and PCI) [22].

Current evidence suggests that surgical revascularization is associated with a significantly lower incidence of major adverse cardiac events, particularly with a reduced rate of acute myocardial infarctions after revascularization [23]. It is also important to underline that the guideline‐directed OMGT was significantly improved and expanded in recent years [24]. Most studies investigating revascularization in ischemic HF patients have been conducted in patients with a median LVEF of approximately 30% [8, 23, 25]. However, patients with LVEF ≤ 20%, who were predominantly represented in our research, experience substantially lower survival rates and higher rehospitalization rates due to decompensated heart failure [5]. These are the patients in whom durable VAD implantation may be considered for long‐term support [5, 6].

## 5 Summary

Our case series highlights that the protected CABG concept is a viable strategy for high‐risk patients with CAD and severely reduced LVEF. Postoperative low cardiac output syndrome remains a feared complication in cardiac surgery, often resulting in devastating outcomes and an in‐hospital mortality rate of approximately 60%, depending on the etiology and the patient's preoperative condition [26]. Early initiation of tMCS in high‐risk patients has been shown to significantly improve outcomes [27].

The on‐pump beating‐heart (ONBEAT) CABG is a crucial alternative to CABG on mAFP, offering enhanced intraoperative support and safety in complex cases. A meta‐analysis has demonstrated that the ONBEAT approach is associated with lower mortality and morbidity compared to cardioplegic CABG [28]. While limited evidence comparing on‐pump and off‐pump CABG suggests higher rates of adverse events with CPB use, the ONBEAT technique enables a greater number of distal anastomoses and facilitates complete revascularization compared to the off‐pump method [29, 30].

Sufficient scientific evidence suggests potential benefits of perioperative prophylactic use of an intra‐aortic balloon pump (IABP) in high‐risk patients undergoing CABG [3, 31]. The advantages of IABP include percutaneous implantation, a small‐bore catheter, and low anticoagulation requirements. IABP improves coronary perfusion during diastole, but provides only limited circulatory support and passive left ventricular (LV) unloading [32]. In contrast, currently available mAFPs are able to provide up to full circulatory support along with active LV unloading [32]. However, this comes at the cost of a higher incidence of therapy‐associated complications that may negatively affect the outcomes [33]. Future studies comparing full‐support mAFP with IABP for perioperative revascularization in CABG patients are of high interest.

In this context, we propose that complete revascularization can be effectively achieved through LV‐unloading with the mAFP. Notably, mAFP provides additional support during the critical early postoperative period, enabling rapid weaning from inotropes and vasopressors, as well as a gradual reintroduction of heart‐failure medications. Axillary cannulation, as performed in our patients, facilitates early mobilization on ongoing support and stabilizes the clinical condition for further evaluation of myocardial function and informed decision‐making regarding durable heart replacement therapies, if necessary [34]. The economic aspects of mAFP application for intra‐ and postoperative tMCS support in our patient population remain a topic of considerable debate. However, if a protected CABG procedure is successfully performed, it allows patients to be weaned from tMCS, thereby potentially avoiding further complications, prolonged hospitalization, or even durable LVAD implantation or death [16].

## 6 Limitations

The small cohort of highly selected patients precluded a robust statistical analysis. A particularly relevant and promising area for further research is the identification of preoperative determinants of myocardial recovery in protected CABG patients.

In the absence of clear evidence for cardiac MRI or viability assessment, advanced echocardiographic evaluation plays a crucial role in therapy decision‐making and perioperative prognosis [4]. Future studies should focus on the precise evaluation of left ventricular dilatation severity and novel echocardiographic parameters, such as strain measurements, which hold significant potential for guiding treatment strategies [12].

## 7 Conclusion

Protected CABG using axillary‐implanted full‐support mAFPs is a feasible approach for treating CAD patients with severely reduced LVEF. This strategy provides intraoperative circulatory support while reducing the risk of postoperative low cardiac output syndrome and may potentially prevent or delay the need for durable LVAD implantation in this patient population. However, therapy‐associated complications, particularly thromboembolic strokes and bleeding, warrant further investigation and optimization. The indications for mAFP application in the setting of protected CABG should be carefully weighed against the risk of therapy‐associated complications. Prospective randomized trials are essential to demonstrate the benefits of intraoperative mAFP support during surgical revascularization.

## Author Contributions

GN: Conceptualization, Methodology, Visualization, Writing – original draft, Writing – review & editing. DL: Writing – review & editing. YH: Writing – review & editing. PL: Writing – original draft, Writing – review & editing. SO: Writing – original draft, Writing – review & editing. NM: Methodology, Data curation, Writing – review & editing. VF: Supervision, Writing – original draft, Writing – review & editing. FS: Conceptualization, Investigation, Methodology, Supervision, Writing – original draft, Writing – review & editing. EP: Conceptualization, Methodology, Supervision, Writing – original draft, Writing – review & editing. AA: Conceptualization, Methodology, Supervision, Writing – original draft, Writing – review & editing.

## Conflicts of Interest

S.O. received institutional research and study funds from Novartis Pharma GmbH and institutional research, study, and educational grants, speaker fees, and advisory board fees from Abiomed outside the submitted work. F.S. received institutional grants from Novartis, Abbott, and institutional fees (speaker honoraria) from Abbott, Bayer, Novartis, and Abiomed outside of the submitted work. V.F. declares relevant financial activities outside the submitted work with the following commercial entities: Medtronic GmbH, Biotronik SE & Co., Abbott GmbH & Co. KG, Boston Scientific, Edwards Lifesciences, Berlin Heart, Novartis Pharma GmbH, JOTEC GmbH, and Zurich Heart in relation to educational grants (including travel support), fees for lectures and speeches, fees for professional consultation, and research and study funds. E.P. declares payment related to proctoring, consulting, speaker fees, and institutional grants from Abbott, Abiomed, Medtronic, Recovery Therapeutics, and Cor Medical outside of the submitted work. P.L. received study and educational grants and speaker fees from Abiomed and Abbott outside the submitted work.

## Data Availability

Presented data is available upon special request to the corresponding author.
